# Brain signal variability and executive functions across the life span

**DOI:** 10.1162/netn_a_00347

**Published:** 2024-04-01

**Authors:** Zachary T. Goodman, Jason S. Nomi, Salome Kornfeld, Taylor Bolt, Roger A. Saumure, Celia Romero, Sierra A. Bainter, Lucina Q. Uddin

**Affiliations:** Department of Psychology, University of Miami, Coral Gables, FL, USA; Semel Institute for Neuroscience and Human Behavior, Department of Psychiatry and Biobehavioral Sciences, University of California Los Angeles, Los Angeles, CA, USA; REHAB Basel, Klinik für Neurorehabilitation und Paraplegiologie, Basel, Switzerland; Department of Psychology, University of California Los Angeles, Los Angeles, CA, USA

**Keywords:** Brain signal variability, Executive functions, Cognitive flexibility, Life span, Resting-state fMRI

## Abstract

Neural variability is thought to facilitate survival through flexible adaptation to changing environmental demands. In humans, such capacity for flexible adaptation may manifest as fluid reasoning, inhibition of automatic responses, and mental set-switching—skills falling under the broad domain of executive functions that fluctuate over the life span. Neural variability can be quantified via the BOLD signal in resting-state fMRI. Variability of large-scale brain networks is posited to underpin complex cognitive activities requiring interactions between multiple brain regions. Few studies have examined the extent to which network-level brain signal variability across the life span maps onto high-level processes under the umbrella of executive functions. The present study leveraged a large publicly available neuroimaging dataset to investigate the relationship between signal variability and executive functions across the life span. Associations between brain signal variability and executive functions shifted as a function of age. Limbic-specific variability was consistently associated with greater performance across subcomponents of executive functions. Associations between executive function subcomponents and network-level variability of the default mode and central executive networks, as well as whole-brain variability, varied across the life span. Findings suggest that brain signal variability may help to explain to age-related differences in executive functions across the life span.

## Author Summary

Traditionally, regional variability in brain signals has been viewed as a source of noise in human neuroimaging research. Our study demonstrates that brain signal variability may contain meaningful information related to psychological processes. We demonstrate that brain signal variability, particularly whole-brain variability, may serve as a reliable indicator of cognitive functions across the life span. Global variability and network-level variability play differing roles in supporting executive functions. Findings suggest that brain signal variability serves as a meaningful indicator of development and cognitive aging.

## INTRODUCTION

The human brain undergoes protracted developmental changes over the lifetime as systems develop and adapt (Spear, 2013). Executive functions (EF) are an important cognitive domain known to fluctuate across the life span (Diamond, 2013). EFs are critical for success in executing goal-directed behaviors, including planning, self-monitoring, sustained attention, mental control, inhibition, or flexibility (Diamond, 2013). Within the broad EF domain, specific cognitive abilities vary in the age at which they grow, peak, and decline (Ferguson et al., 2021). By the end of early adulthood, mild declines can already be observed in processing speed, visuospatial reasoning, and learning and memory (Ferguson et al., 2021; Hartshorne & Germine, 2015; Salthouse, 2009).

As a marker of neural flexibility (Garrett, Samanez-Larkin, et al., 2013; Nomi et al., 2017), brain signal variability may underlie a variety of fluid cognitive functions that develop throughout adolescence, are formalized by early adulthood, and gradually decline throughout middle and later adulthood (Finkel et al., 2007; McArdle et al., 2002). Brain signal variability has been shown to capture important age-related effects in the brain (Garrett et al., 2010; Misić et al., 2010; Nomi et al., 2017). One of the earliest studies using fMRI to study brain signal variability found that between the ages of 20 and 85, the majority of brain voxels decreased in variability with age (Garrett et al., 2010). Younger, faster, and more consistent performers exhibit higher brain signal variability compared with older, poorer performing adults (Garrett et al., 2011). In a longitudinal study, healthy adults who lost brain signal variability over a 2.5-year period also declined in cognition; those who maintained or increased variability also maintained or increased their levels of cognitive performance (Garrett et al., 2021). Additionally, those expressing greater variability loss also exhibited greater decline in cognition (Garrett et al., 2021).

Older age has previously been associated with decreased cortical variability (Garrett, Kovacevic, et al., 2013) and increased subcortical variability (Samanez-Larkin et al., 2010). More specifically, aging corresponds with a linear decrease in variability of the sensorimotor, default-mode, central executive, and visual networks, but a linear increase in salience network variability (Nomi et al., 2017). Overall, higher brain signal variability has been associated with a greater degree of cognitive efficiency, flexibility, set shifting, psychomotor speed, and attentional control throughout adulthood (Garrett et al., 2011; Garrett, Kovacevic, et al., 2013; Grady & Garrett, 2014). In summary, brain signal variability may be indicative of brain network development, organization, flexibility, and integrity across the life span. However, the degree to which brain signal variability corresponds to high-level cognitive faculties and the extent to which such associations vary across the life span and across brain networks both remain undetermined.

The salience network is thought to modulate other brain networks including the central executive and default mode networks (Uddin, 2015). Based on previous work (Nomi et al., 2017), we hypothesized that lower levels of salience network variability in early life and greater levels of salience network variability in later life may adversely impact network switching. During early to mid-life, variability of the salience network may be optimal for the maintenance and switching of networks relevant to cognitive task demands.

We test this hypothesis by leveraging a large life span sample to elucidate associations between brain signal variability and multiple EF tasks spanning cognitive flexibility, processing speed, verbal fluency, working memory, and attention. Previous studies relating brain signal variability to EF skills are limited in the breadth of cognitive tasks or the age period considered. The present study sought to fill these gaps and to demonstrate how brain signal variability underlies EF across the life span.

## MATERIALS AND METHODS

### Participants

Neuroimaging and phenotypic data were obtained from the publicly available Enhanced Nathan Kline Institute database (Nooner et al., 2012). Participants were assessed over 1 to 2 days, during which they completed a battery of neuropsychological assessments, self-report questionnaires, clinical interviews, and MRI scans. The current sample was selected according to the following inclusion criteria: availability of neuroimaging and behavioral data, passing of MRI image quality checks, and average framewise displacement less than 0.5 mm (Power et al., 2012). After exclusions, the final sample consisted of 724 participants (57% female; 10% left-handed), ranging from 6 to 85 years of age (*M* = 35.19, *SD* = 20.81). Nineteen percent of participants identified as African American, 15% as Hispanic, 7% as Asian American/Pacific Islander, and 4% as another ethnicity.

### Image Acquisition and Preprocessing

Images were obtained on a Siemens Trio 3.0T scanner that collected T1 anatomical images (MPRAGE) and a 10-min multiband (x4) EPI sequenced resting-state images (TR = 1.40 s, 2 mm^3^, 64 interleaved slices, TE = 30 ms, FA = 65°, FOV = 224 mm). Participants were instructed to keep their eyes open and to fixate on a cross in the center of the screen (MRI protocol: https://fcon_1000.projects.nitrc.org/indi/enhanced/mri_protocol.html). Images were subjected to visual quality control by six trained research assistants to identify scanner and motion artifacts (e.g., signal loss, inadequate head coverage, warping). Each scan was independently rated as pass or fail by two research assistants, and scans with two failed ratings were excluded. Disagreements were adjudicated by one of the senior neuroscientists.

Resting-state fMRI data that passed quality control were then preprocessed via DPABI (Yan et al., 2016). Steps included the removal of the first five time points, realignment, normalization, and smoothing (AFNI’s 3dBlur algorithm). Subject-level independent component analysis (ICA) was conducted via MELODIC in FSL. Individual components were extracted for 24 subjects at every 10-year interval across all ages, classified into signal or noise components, and regressed from subject space via the ICA-FIX classification algorithm (Griffanti et al., 2014). Following ICA denoising, the data underwent regression of noise covariates (Friston 24 motion parameters) and bandpass filtering (0.01–0.10 Hz).

The Human Brainnetome Atlas (Fan et al., 2016) was selected for brain parcellation because of concordance with functional brain networks and representation of subcortical regions. We derived a total of 246 cortical and subcortical/limbic nuclei regions of interest (ROIs) across five networks: the central executive (CEN), default mode (DMN), salience (SN), limbic (LN), and dorsal attention (DN) networks. ROIs were organized into brain networks based upon alignment with functionally derived brain parcellations (Supplemental Figure 1 in the Supporting Information).

### Neuropsychological Assessment of Executive Functions

#### Delis-kaplan executive function system.

The Delis-Kaplan Executive Function System (D-KEFS; Delis et al., 2001) is a compilation of neuropsychological tests assessing multiple cognitive functions. Three tests were selected to represent a range of cognitive functions. The Trail Making Test (TMT) measures processing speed, cognitive flexibility, and set shifting. The TMT requires participants to sequentially connect circles filled with numbers or letters. Three scores were calculated: TMT-A, average time-to-completion across the number sequencing and letter sequencing subtests; TMT-B, time-to-completion on the number-letter switching subtest; and TMT-MS, time-to-completion on the motor speed subtest. Two trials of the Color-Word Interference (CWI) Test were included. The *inhibition* (CWI-I) trial requires participants to state the color of printed words (e.g., responding “red” when RED is printed in blue ink), while the *switching* (CWI-S) trial requires participants to alternate between stating the written word and the color of ink. Performance for each trial is measured as the seconds required to complete the sheet of words. *Verbal fluency* includes a series of 1-min trials requiring the participant to generate a number of words. The *letter fluency* (LF) subtest includes three trials in which participants generate words based on a beginning letter. The *category fluency* (CF) subtest includes two trials in which generated words are based on semantic categories. *Category switching* (CS) requires word generation from alternating semantic categories (fruits and furniture), measuring both verbal fluency and cognitive flexibility. CS scores were recorded as the number of correct switches between categories. The Card Sorting Test (CST) requires participants to match stimulus cards based on either visuospatial or semantic properties. The number of correct sorts was used as the primary score.

#### Penn computerized neurocognitive battery.

Select tasks of the Penn Computerized Neurocognitive Battery (CNB; Gur et al., 2010) were included to supplement available D-KEFS scores. The CNB is a brief cognitive battery assessing attention, working memory, processing speed, and reasoning skills that has been validated across the life span (Gur et al., 2010, 2012). The Penn Conditional Exclusion Test (PCET; Kurtz et al., 2004), the Penn Continuous Performance Test (CPT; Kurtz et al., 2001), and the *letter n-back* (LNB; Ragland et al., 2002) tasks were included to assess processing speed and working memory, respectively. The PCET is an “odd one out” task requiring participants to identify which of four objects does not belong based on visual properties, with performance determined as average reaction time across 10 trials. The CPT requires participants to respond when presented with a target number sequence, with performance measured as average reaction time across 30 target sequences over 360 trials. The LNB includes three conditions—0-back, 1-back, and 2-back—each of which presents letters one after another, with correct responses based on a rule per condition: when the letter X is displayed (0-back); when the current letter matches the prior letter (1-back); and when the current letter matches the letter presented before the prior letter (2-back).

## EXPERIMENTAL DESIGN AND STATISTICAL ANALYSIS

### Calculation of Brain Signal Variability

Within-person brain signal variability was calculated based on the root mean square of successive differences (rMSSD; von Neumann et al., 1941) for each ROI across the time series. Compared with traditional metrics of variance including standard deviation, which is agnostic to the temporal ordering of data, rMSSD denotes the average deviance of the time series from one moment to the next. The rMSSD scores are reliable metrics of BOLD signal variability (Nomi et al., 2019) and minimize the impact of autocorrelations, which occur often in multiband EPIs (Smith et al., 2013). ROI-wise rMSSD (*δ*_*i*_) is calculated by squaring the sum of successive differences in ROI signal, dividing by number of time points minus 1, and taking the square root:

$$\delta_{i}=\sqrt{\frac{\sum {\left(x_{i_{\left(t-1\right)}}-x_{i_{\left(t\right)}}\right)}^{2}}{\left(n-1\right)}}.$$

Each participant’s head motion was then regressed from *δ*_*i*_. Whole-brain variability was calculated as the mean *δ*_*i*_ across all represented brain regions, while network-level variability was calculated as the mean *δ*_*i*_ across ROIs representing each network.

### Factor Models

Confirmatory factor analysis (CFA) was used to model latent variables of executive function domains. CFA was used to verify the construct validity of executive function subcomponents (i.e., factors) and to reduce the number of regression models necessary. A correlated four-factor model was proposed to represent the set of cognitive tasks: (a) *cognitive flexibility*, indicated by CST, LF, CF, TMT-B, and CS; (b) *inhibitory control*, indicated by CWI-I and CWI-S; (c) *processing speed*, indicated by TMT-A, TMT-MS, and RT; and (d) *working memory*, indicated by 0-back, 1-back, 2-back, and CPT. In the case of suboptimal model fit, as determined by a CFI < 0.95, RMSEA > 0.08, and SRMR > 0.06, covariances between indicators were included based on residuals and modification indices, so long as the indicators (a) were drawn from the same cognitive task and (b) represented the same latent construct. Factor scores were predicted via the regression method (i.e., factor loadings act as weights of a linear model).

### Associations With Cognitive Tasks

Relationships between rMSSD, age, and EF components were tested with linear regression, with factor scores serving as outcomes. All linear models included sex/gender, handedness, and years of education as covariates. Reported *p* values were adjusted for multiple comparisons based on the false discovery rate (Benjamini & Hochberg, 1995). Models were built in a hierarchical fashion, starting with models considering only the main effects of network variability (i.e., rMSSD) on each task. To examine differences in the association between rMSSD and executive functions, interaction terms between age and each network variability metric were included and examined. Statistical tests were based upon interaction terms between continuous covariates (i.e., age and variability). Age-by-variability interaction terms that remained statistically significant after correction for multiple comparisons were then probed at 20-year intervals and the respective conditional slopes were visualized (i.e., “simple slopes”) for four age ranges: adolescence (*M*_age_ = 13.17, *SD*_age_ = 3.23, *n* = 209), early adulthood (*M*_age_ = 27.01, *SD*_age_ = 5.56, *n* = 238), middle adulthood (*M*_age_ = 50.09, *SD*_age_ = 5.74, *n* = 159), and older adulthood (*M*_age_ = 69.42, *SD*_age_ = 6.64, *n* = 121)—the specific intervals chosen for simple slopes are largely arbitrary and serve to facilitate interpretations (Supplemental Figure 2). Conditional slope coefficients and *p* values are reported in the Supporting Information. Age was mean-centered to reduce the impact of collinearity resulting from interaction terms. Standardized coefficients (β) are reported to represent effect sizes. Analyses were conducted in *R* version 4.2.0.

## RESULTS

**Figure 1.**
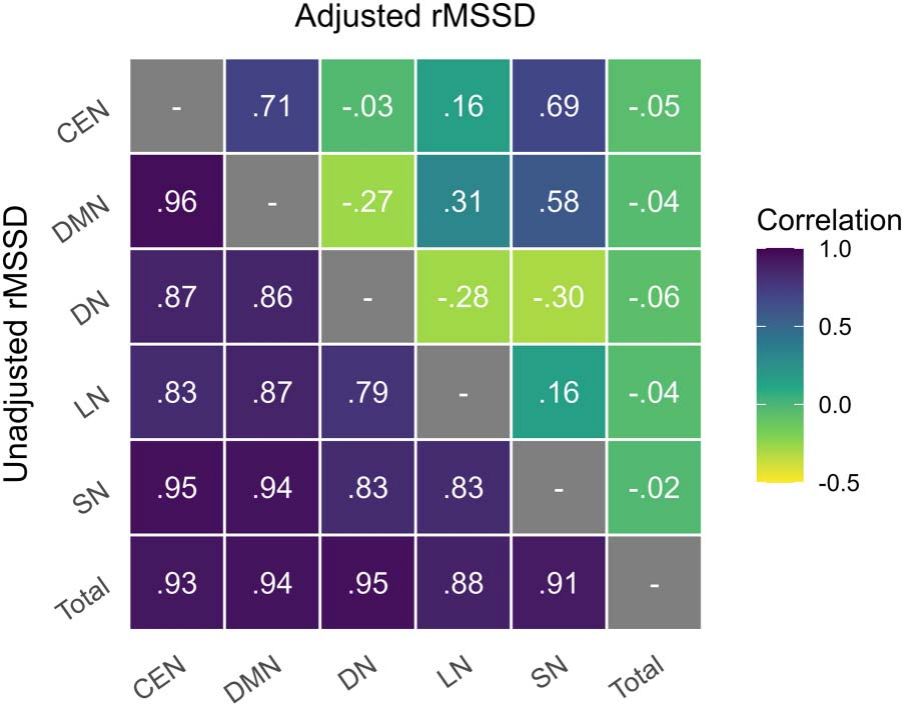
Correlations between network variability before (lower triangle) and after (upper triangle) adjustment for whole-brain variability.

Network-wise correlations in brain signal variability were strong (*r* ≥ 0.79), and all networks were highly correlated with whole-brain variability (Figure 1). Inter-network collinearity exceeded acceptable cutoffs to the extent that simultaneous inclusion would produce unreliable regression estimates and spurious inferences (McClelland et al., 2017). Thus, we propose that a person-wise adjustment of rMSSD is necessary to partial whole-brain variability from signal variability specific to brain networks. Our proposed adjustment is to regress whole-brain rMSSD from each voxel prior to calculating ROI or network-specific signal variability. In a manner similar to whole-brain global signal regression, whole-brain rMSSD regression calculated the average whole-brain difference across all voxels at each time point before regressing it out of each individual voxel-wise time point difference. As shown in Figure 1, applying this adjustment increased network orthogonality, and correlations between adjusted networks ranged from strong and positive to weak and negative. The CEN, DMN, and SN remained highly and positively correlated while the LN and DN were modestly correlated with other networks. After adjustment, whole-brain variability was uncorrelated with variability of all networks.

### Latent Variable Model of Executive Functions

**Figure 2.**
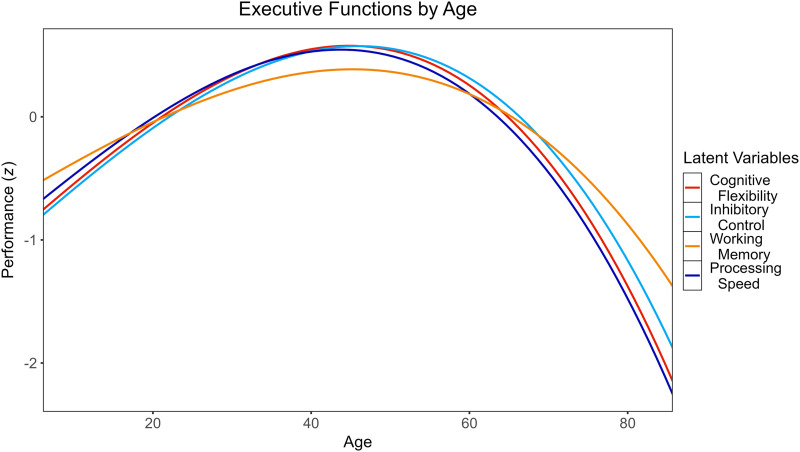
Polynomial relationships between executive function latent variables and age.

Descriptive statistics for each executive functioning test are reported in Table 1. The proposed model demonstrated marginal fit to the data (*χ*^2^(71) = 489.69, *p* < 0.001, CFI = 0.88, RMSEA = 0.10 [0.09, 0.11], SRMR = 0.07). Inclusion of select residual covariances between indicators of the same factor that were drawn from the same executive functioning tasks (e.g., LF and CF) improved fit to an acceptable degree (*χ*^2^(67) = 252.44, *p* < 0.001, CFI = 0.94, RMSEA = 0.07 [0.06, 0.08], SRMR = 0.06). Factor loadings and covariances are reported in Supplemental Table 1. Factor scores were extracted from the modified model and used as primary outcomes in further analyses. Across all executive function latent variables, there were nonlinear associations between EF performance and age (Supplemental Table 2), such that performance generally improved from adolescence into middle adulthood and declined in later adulthood (Figure 2).

**Table 1.** Demographic and descriptive statistics

	*M*	*SD*
Age	35.2	(20.8)
Head motion	0.25	(0.1)
Years of education	12.9	(4.9)
TMT-A (*s*)	32.3	(15.2)
TMT-B (*s*)	83.2	(40.5)
TMT-MS (*s*)	32.3	(15.1)
Letter fluency	37.1	(12.8)
Category fluency	39.9	(9.6)
Category switching	13.3	(3.2)
Card sorting	18.5	(5.4)
CWI – inhibition (*s*)	57.8	(19.9)
CWI – switching (*s*)	64.0	(18.6)
Reaction time (*s*)	2.3	(1.1)
0-back (*ms*)	467	(81)
1-back (*ms*)	506	(151)
2-back (*ms*)	542	(171)
Medical conditions		%
Hypertension		17.0
Hyperlipidemia		25.1
Hypotension		7.9
Diabetes (type II)		3.6
Coronary artery disease		1.5

### Network Variability and Executive Functions

Across all models, limbic network variability was negatively associated with executive functioning (Table 2). In other words, individuals with lower brain signal variability in limbic regions demonstrated higher cognitive flexibility, inhibitory control, processing speed, and working memory. No other associations between network variability and executive functioning factors survived corrections for multiple comparisons.

**Table 2.** Linear regression coefficients for models predicting cognitive performance from network signal variability without interactions (i.e., “main effects”) and network-by-age interaction terms. Bolded values indicate significant effects (*p* < 0.05) surviving corrections for multiple comparisons.

Predictors	Cognitive flexibility			Inhibition			Processing speed			Working memory		
	b	*SE*	*p*	b	*SE*	*p*	b	*SE*	*p*	b	*SE*	*p*
Main effects												
Total	0.02	0.04	0.705	0.00	0.04	0.928	0.04	0.04	0.392	0.04	0.05	0.549
CEN	0.02	0.06	0.745	−0.01	0.06	0.928	0.01	0.06	0.905	0.03	0.06	0.581
DMN	0.11	0.05	0.107	0.12	0.05	0.074	0.12	0.05	0.071	0.04	0.05	0.549
DA	−0.03	0.04	0.563	−0.02	0.04	0.928	−0.05	0.04	0.392	0.03	0.04	0.549
LN	**−0.15**	**0.04**	**<0.001**	**−0.14**	**0.04**	**<0.001**	**−0.14**	**0.04**	**<0.001**	**−0.12**	**0.04**	**0.008**
SN	−0.05	0.05	0.554	−0.04	0.05	0.818	−0.05	0.05	0.392	−0.07	0.05	0.420
Interactions												
Total × age	**0.01**	**<0.01**	**0.001**	**0.01**	**<0.01**	**0.001**	**0.01**	**<0.01**	**0.003**	<0.01	<0.01	0.414
CEN × age	**−0.01**	**<0.01**	**0.031**	**−0.01**	**<0.01**	**0.036**	**−0.01**	**<0.01**	**0.038**	**−0.01**	<0.01	**0.011**
DMN × age	**0.01**	**<0.01**	**0.002**	**0.01**	**<0.01**	**0.006**	**0.01**	**<0.01**	**0.003**	**0.01**	<0.01	**0.011**
DA × age	<0.01	<0.01	0.997	<0.01	<0.01	0.529	<0.01	<0.01	0.881	<0.01	<0.01	0.930
LN × age	<0.01	<0.01	0.268	<0.01	<0.01	0.529	<0.01	<0.01	0.343	<0.01	<0.01	0.930
SN × age	<0.01	<0.01	0.690	<0.01	<0.01	0.529	<0.01	<0.01	0.667	<0.01	<0.01	0.930

### Network Variability Across the Life Span

Several network variability-by-age interactions emerged (Table 2). Interactions between age and both DMN and CEN variability were significant across all executive functioning domains. The interaction between age and whole-brain variability was significant for cognitive flexibility, inhibitory control, and processing speed, but not working memory. Simple slope coefficients are reported in Supplemental Table 3.

**Figure 3.**
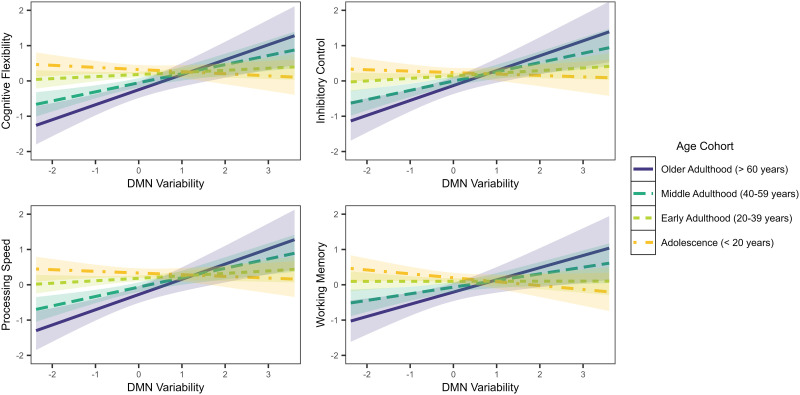
Associations between executive functions and DMN-specific brain signal variability by age cohort.

**Figure 4.**
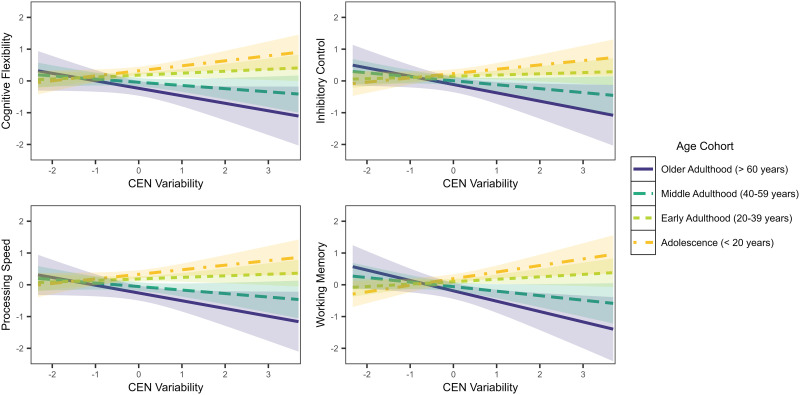
Associations between executive functions and CEN-specific brain signal variability by age cohort.

The significant interaction between age and DMN-specific variability was similar across cognitive tasks—variability was unassociated with executive functions in adolescence and early adulthood, and positively associated with executive functions in middle to older adulthood (Figure 3). An opposite pattern emerged with respect to the interaction between age and CEN-specific variability. The association between CEN variability and executive functions was positive in adolescence and negative in later adulthood (Figure 4).

**Figure 5.**
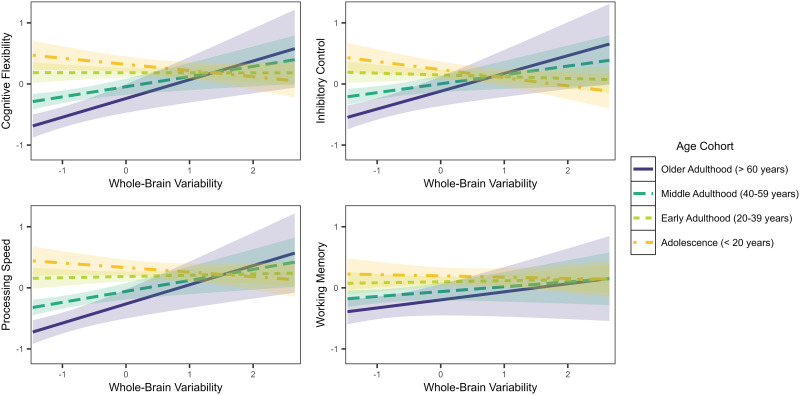
Associations between executive functions and whole-brain signal variability by age cohort.

Lastly, whole-brain variability demonstrated a pattern more similar to DMN-specific variability across the life span. Associations between whole-brain variability and *cognitive flexibility* and *processing speed* were nonsignificant in younger adulthood, but significant and positive in middle and older adulthood (Figure 5). The association was significant and negative in younger adulthood, and significant and positive, in *inhibitory control* only. The interaction between whole-brain variability and working memory was not significant.

## DISCUSSION

The present study leveraged a large community sample to investigate relationships between brain signal variability and EF, and the extent to which such relationships fluctuate across the life span. To date, brain signal variability as a metric of individual differences in high-level cognition has received relatively little attention within cognitive neuroscience (Garrett, Kovacevic, et al., 2013), but may nonetheless be a necessary property underlying adaptation to changing environments (Garrett et al., 2011; Garrett, Samanez-Larkin, et al., 2013), functional brain connectivity (Uddin, 2020), and cognitive efficiency (Garrett, Samanez-Larkin, et al., 2013). Consistent with this theory, our findings demonstrated a set of relationships between components of executive functions and brain signal variability.

Whole-brain signal variability was highly interrelated to the variability of intrinsic brain networks. Further, observed correlations between network-specific variabilities were very strong. For example, the unadjusted default mode and central executive networks share 92% of their variances. This suggests that a high degree of overlap exists, which is statistically problematic. Our adjustment to regional signal variability resulted in variabilities of intrinsic brain networks that are nearly orthogonal to whole-brain variability and are more appropriately interrelated. After adjustments, the default mode and central executive networks share 50% of variance. As a result, task-based relationships with whole-brain variability represent differences in cognitive functions associated with global variability, whereas task-based relationships with network-specific variabilities represent differences in cognitive functions associated with brain networks above and beyond that which is attributable to whole-brain variability.

Associations between whole-brain variability and executive functions were most compelling in the older adulthood cohort, for which greater whole-brain variability appeared to facilitate better cognitive flexibility, set shifting, and processing speed. These findings are consistent with indications that changes in regional brain signal variability are related to age-related declines in reaction time (Guitart-Masip et al., 2016), and may help to explain cognitive aging more broadly (Garrett et al., 2017; Grady & Garrett, 2014). In the adolescent cohort, effects of whole-brain variability were milder, with greater variability appearing to interfere with inhibitory control but demonstrating no relationship to cognitive flexibility, processing speed, or working memory. The apparent benefit of global signal variability in older adulthood may reflect compensatory brain mechanisms leveraged during the aging process, in which it is proposed that recruitment of a greater number of brain regions is required to offset degradation of systems that were more localized earlier in life (Spreng & Turner, 2019). Brain system modulation is required to successfully navigate complex cognitive tasks (Rieck et al., 2017), and greater signal variability in aging may indicate preservation of such capacities. In contrast, brain networks become increasingly integrated and localized throughout adolescence (Fair et al., 2009). The present study suggests that greater-than-average whole-brain variability may represent delayed integration or interference with network-specific functions, at least in younger individuals.

Irrespective of aging, limbic variability was consistently associated with poorer cognitive flexibility, inhibitory control, processing speed, and working memory. In older adulthood, DMN hyper-variability was correlated with greater executive functions, across subcomponents. This finding maps onto a broader connectivity literature that has revealed disinhibition of the DMN may impair cognition (Anticevic et al., 2012). Moreover, it may be the case that greater regional brain signal variability of the DMN corresponds to maintained inhibition of the DMN during cognitive task performance, preserving executive functions. Conversely, central executive network hyper-variability seemed deleterious to those in older adulthood but had no effect in adolescence to early adulthood.

Regional changes in variability may be one explanation for changes in connectivity and associated cognitive decline that is noted to occur in mid- to late adulthood (Finkel et al., 2007; Grady et al., 2006); however, the exact associations between the current findings with functional connectivity strength is unclear. Previous research in adult resting-state fMRI data has shown that within-network connectivity is associated with greater brain signal variability similarity between brain regions compared with between-network connectivity that has lower brain signal variability similarity between brain regions (Baracchini et al., 2021). Additionally, higher brain signal variability similarity between regions is associated with stronger functional connectivity strength compared with functionally connected regions with lower brain signal variability. It was also shown that variability-connectivity relationships followed a unimodal-transmodal gradient differentiating between lower order sensory regions and higher order cognitive regions in accord with previous research on functional connectivity gradients in humans and macaques (Margulies et al., 2016). This suggests an intimate relationship between brain structure, BOLD signal variability, and functional connectivity; as brain structure and functional connectivity changes across the life span, one would surmise that this also impacts variability-connectivity relationships.

The current results show that increased within-network variability can be helpful in aging, as in the case of the DMN or whole-brain variability where increased variability in older adults was positively related to EF ability. The current results also show that increased variability in older adults can be detrimental to EF ability, as in the case of the CEN. In the context of maturational changes in functional connectivity, both of these networks would be hypothesized to show decreased within-network connectivity (Betzel et al., 2016; Vij et al., 2018) despite showing differences in variability-EF associations. Thus, it is unclear how functional connectivity strength would play a role in the relationships presented here. Further, it is unknown how between-network connectivity, which typically increases in age (Betzel et al., 2016; Vij et al., 2018), is related to the current variability-EF associations, as quantifying between-network variability across different networks is not straightforward. That is, it is unclear how one would want to average variability across other networks in the brain when focusing on a specific network such as the DMN or CEN. Future research is needed in order to quantify the association between variability, connectivity, age, and executive function in order to determine how functional connectivity strength plays a role in the relationships described here.

There are several limitations of note. Previous longitudinal research has shown that decreases in an individual’s BOLD variability across time is related to decreases in cognitive performance, while individuals who show little change in BOLD signal variability across time show no decreases and sometimes even an increase in cognitive performance; such relationships were most prominent within the fronto-striato-thalamic system (Garrett et al., 2021). As the current study aimed at exploring canonical large-scale neural networks such as the DMN and CEN, future research should examine how variability in subcortical brain areas such as the thalamus and striatum may be associated with EF performance across the life span. Additionally, an important consideration in any study exploring brain function across the life span is accounting for physiological changes such as vascular or cardiac processes that are known to influence brain activity and cognitive function (Jacobs et al., 2013; Son et al., 2015; Waldstein et al., 2005); however, previous BOLD signal variability research has shown that life span aging effects still persist and remain robust after carefully controlling for vascular confounds (Garrett et al., 2017), ameliorating this concern within the current study. Lastly, research on the quantification and relevance of brain signal variability is nascent, particularly within the resting-state fMRI literature. Therefore, reproducibility in independent samples, across cohorts and measures of executive functions, is necessary to validate the implications of this study. Similarly, the reliability of brain signal variability and the stability of associations between brain signal variability and cognitive functions across the life span are both open questions.

To our knowledge, this study is the first to investigate relationships between global and network-level brain signal variability and a range of executive functions across the life span. Broadly, these findings align with the general notion that age-based fluctuations in executive functions may be attributable to both regional and global changes in brain functional integrity (Best & Miller, 2010; Diamond, 2013; Kupis et al., 2021; Nashiro et al., 2017). Moreover, this study illustrates that the relationship between brain signal variability and behavioral task performance is not constant across the life span. Further research is warranted to understand the underlying mechanisms that give rise to interindividual differences in brain signal variability, as well as mechanisms through which brain signal variability is expressed behaviorally.

## DATA AVAILABILITY

Materials necessary to reproduce these analyses are available at the Open Science Framework: https://osf.io/zw2bv/. Data are available through the Nathan Kline Institute.

## SUPPORTING INFORMATION

Supporting information for this article is available at https://doi.org/10.1162/netn_a_00347.

## AUTHOR CONTRIBUTIONS

Zachary Goodman: Conceptualization; Data curation; Formal analysis; Methodology; Visualization; Writing – original draft; Writing – review & editing. Jason S. Nomi: Conceptualization; Funding acquisition; Investigation; Methodology; Project administration; Supervision; Writing – review & editing. Salome Kornfeld: Conceptualization; Data curation; Formal analysis; Writing – original draft. Taylor Bolt: Methodology; Writing – review & editing. Roger A. Saumure: Formal analysis; Software. Celia Romero: Resources; Writing – review & editing. Sierra A. Bainter: Formal analysis; Supervision; Writing – review & editing. Lucina Q. Uddin: Conceptualization; Funding acquisition; Investigation; Project administration; Supervision; Writing – review & editing.

## FUNDING INFORMATION

Zachary Goodman, National Institutes of Health (https://dx.doi.org/10.13039/100000002), Award ID: T32-HL007426. Jason S. Nomi, National Institute of Mental Health (https://dx.doi.org/10.13039/100000025), Award ID: R03-MH121668. Salome Kornfeld, Freiwillige Akademische Gesellschaft (https://dx.doi.org/10.13039/100009736). Jason S. Nomi, National Alliance for Research on Schizophrenia and Depression (https://dx.doi.org/10.13039/100009670). Sierra A. Bainter, National Institute of Mental Health (https://dx.doi.org/10.13039/100000025), Award ID: K01-MH122805. Lucina Q. Uddin, National Institute of Mental Health (https://dx.doi.org/10.13039/100000025), Award ID: R01-MH107549. Lucina Q. Uddin, Canadian Institute for Advanced Research (https://dx.doi.org/10.13039/100007631). Lucina Q. Uddin, University of Miami Gabelli Senior Scholar Award.

## ETHICS STATEMENT

This study was approved by the University of Miami Institutional Review Board.

## Supplementary Material



## TECHNICAL TERMS

Brain signal variability: A measure of signal irregularity, sometimes computed using the mean successive squared difference (MSSD) of BOLD signal intensities across data points.
Mean successive squared difference (MSSD): A metric often used to quantify brain signal variability in which both the magnitude of distance and the temporal ordering of data are considered.
Whole-brain variability: Individual subject-level estimates of average variability across all cortical regions.
Network-specific variability: Variability of specific brain networks, computed by partialling whole-brain variability from each voxel prior to calculation.
Confirmatory factor analysis: An analytic framework for testing the statistical fit of theoretical latent variables.
Latent variables: Constructs not amenable to direct measurement, but inferred from a set of observed variables.

## References

[bib1] Anticevic, A., Cole, M. W., Murray, J. D., Corlett, P. R., Wang, X.-J., & Krystal, J. H. (2012). The role of default network deactivation in cognition and disease. Trends in Cognitive Sciences, 16(12), 584–592. 10.1016/j.tics.2012.10.008, 23142417 PMC3501603

[bib2] Baracchini, G., Mišić, B., Setton, R., Mwilambwe-Tshilobo, L., Girn, M., Nomi, J. S., Uddin, L. Q., Turner, G. R., & Spreng, R. N. (2021). Inter-regional BOLD signal variability is an organizational feature of functional brain networks. NeuroImage, 237, 118149. 10.1016/j.neuroimage.2021.118149, 33991695 PMC8970039

[bib3] Benjamini, Y., & Hochberg, Y. (1995). Controlling the false discovery rate: A practical and powerful approach to multiple testing. Journal of the Royal Statistical Society: Series B (Methodological), 57(1), 289–300. 10.1111/j.2517-6161.1995.tb02031.x

[bib4] Best, J. R., & Miller, P. H. (2010). A developmental perspective on executive function. Child Development, 81(6), 1641–1660. 10.1111/j.1467-8624.2010.01499.x, 21077853 PMC3058827

[bib5] Betzel, R. F., Avena-Koenigsberger, A., Goñi, J., He, Y., de Reus, M. A., Griffa, A., Vértes, P. E., Mišic, B., Thiran, J.-P., Hagmann, P., van den Heuvel, M., Zuo, X.-N., Bullmore, E. T., & Sporns, O. (2016). Generative models of the human connectome. NeuroImage, 124(Pt. A), 1054–1064. 10.1016/j.neuroimage.2015.09.041, 26427642 PMC4655950

[bib6] Delis, D. C., Kaplan, E., & Kramer, J. H. (2001). Delis-Kaplan executive function system: Technical manual. The Psychological Corporation. 10.1037/t15082-000

[bib7] Diamond, A. (2013). Executive functions. Annual Review of Psychology, 64, 135–168. 10.1146/annurev-psych-113011-143750, 23020641 PMC4084861

[bib8] Fair, D. A., Cohen, A. L., Power, J. D., Dosenbach, N. U. F., Church, J. A., Miezin, F. M., Schlaggar, B. L., & Petersen, S. E. (2009). Functional brain networks develop from a “local to distributed” organization. PLOS Computational Biology, 5(5), e1000381. 10.1371/journal.pcbi.1000381, 19412534 PMC2671306

[bib9] Fan, L., Li, H., Zhuo, J., Zhang, Y., Wang, J., Chen, L., Yang, Z., Chu, C., Xie, S., Laird, A. R., Fox, P. T., Eickhoff, S. B., Yu, C., & Jiang, T. (2016). The Human Brainnetome Atlas: A new brain atlas based on connectional architecture. Cerebral Cortex, 26(8), 3508–3526. 10.1093/cercor/bhw157, 27230218 PMC4961028

[bib10] Ferguson, H. J., Brunsdon, V. E. A., & Bradford, E. E. F. (2021). The developmental trajectories of executive function from adolescence to old age. Scientific Reports, 11(1), 1382. 10.1038/s41598-020-80866-1, 33446798 PMC7809200

[bib11] Finkel, D., Reynolds, C. A., McArdle, J. J., & Pedersen, N. L. (2007). Age changes in processing speed as a leading indicator of cognitive aging. Psychology and Aging, 22(3), 558–568. 10.1037/0882-7974.22.3.558, 17874954

[bib12] Garrett, D. D., Kovacevic, N., McIntosh, A. R., & Grady, C. L. (2010). Blood oxygen level-dependent signal variability is more than just noise. Journal of Neuroscience, 30(14), 4914–4921. 10.1523/JNEUROSCI.5166-09.2010, 20371811 PMC6632804

[bib13] Garrett, D. D., Kovacevic, N., McIntosh, A. R., & Grady, C. L. (2011). The importance of being variable. Journal of Neuroscience, 31(12), 4496–4503. 10.1523/JNEUROSCI.5641-10.2011, 21430150 PMC3104038

[bib14] Garrett, D. D., Kovacevic, N., McIntosh, A. R., & Grady, C. L. (2013). The modulation of BOLD variability between cognitive states varies by age and processing speed. Cerebral Cortex, 23(3), 684–693. 10.1093/cercor/bhs055, 22419679 PMC3823571

[bib15] Garrett, D. D., Lindenberger, U., Hoge, R. D., & Gauthier, C. J. (2017). Age differences in brain signal variability are robust to multiple vascular controls. Scientific Reports, 7(1), 10149. 10.1038/s41598-017-09752-7, 28860455 PMC5579254

[bib16] Garrett, D. D., Samanez-Larkin, G. R., MacDonald, S. W. S., Lindenberger, U., McIntosh, A. R., & Grady, C. L. (2013). Moment-to-moment brain signal variability: A next frontier in human brain mapping? Neuroscience and Biobehavioral Reviews, 37(4), 610–624. 10.1016/j.neubiorev.2013.02.015, 23458776 PMC3732213

[bib17] Garrett, D. D., Skowron, A., Wiegert, S., Adolf, J., Dahle, C. L., Lindenberger, U., & Raz, N. (2021). Lost dynamics and the dynamics of loss: Longitudinal compression of brain signal variability is coupled with declines in functional integration and cognitive performance. Cerebral Cortex, 31(11), 5239–5252. 10.1093/cercor/bhab154, 34297815 PMC8491679

[bib18] Grady, C. L., & Garrett, D. D. (2014). Understanding variability in the BOLD signal and why it matters for aging. Brain Imaging and Behavior, 8(2), 274–283. 10.1007/s11682-013-9253-0, 24008589 PMC3922711

[bib19] Grady, C. L., Springer, M. V., Hongwanishkul, D., McIntosh, A. R., & Winocur, G. (2006). Age-related changes in brain activity across the adult lifespan. Journal of Cognitive Neuroscience, 18(2), 227–241. 10.1162/jocn.2006.18.2.227, 16494683

[bib20] Griffanti, L., Salimi-Khorshidi, G., Beckmann, C. F., Auerbach, E. J., Douaud, G., Sexton, C. E., Zsoldos, E., Ebmeier, K. P., Filippini, N., Mackay, C. E., Moeller, S., Xu, J., Yacoub, E., Baselli, G., Ugurbil, K., Miller, K. L., & Smith, S. M. (2014). ICA-based artefact removal and accelerated fMRI acquisition for improved resting state network imaging. NeuroImage, 95, 232–247. 10.1016/j.neuroimage.2014.03.034, 24657355 PMC4154346

[bib21] Guitart-Masip, M., Salami, A., Garrett, D., Rieckmann, A., Lindenberger, U., & Bäckman, L. (2016). BOLD variability is related to dopaminergic neurotransmission and cognitive aging. Cerebral Cortex, 26(5), 2074–2083. 10.1093/cercor/bhv029, 25750252

[bib23] Gur, R. C., Richard, J., Calkins, M. E., Chiavacci, R., Hansen, J. A., Bilker, W. B., Loughead, J., Connolly, J. J., Qiu, H., Mentch, F. D., Abou-Sleiman, P. M., Hakonarson, H., & Gur, R. E. (2012). Age group and sex differences in performance on a computerized neurocognitive battery in children age 8–21. Neuropsychology, 26(2), 251–265. 10.1037/a0026712, 22251308 PMC3295891

[bib22] Gur, R. C., Richard, J., Hughett, P., Calkins, M. E., Macy, L., Bilker, W. B., Brensinger, C., & Gur, R. E. (2010). A cognitive neuroscience-based computerized battery for efficient measurement of individual differences: Standardization and initial construct validation. Journal of Neuroscience Methods, 187(2), 254–262. 10.1016/j.jneumeth.2009.11.017, 19945485 PMC2832711

[bib24] Hartshorne, J. K., & Germine, L. T. (2015). When does cognitive functioning peak? The asynchronous rise and fall of different cognitive abilities across the lifespan. Psychological Science, 26(4), 433–443. 10.1177/0956797614567339, 25770099 PMC4441622

[bib25] Jacobs, H. I. L., Leritz, E. C., Williams, V. J., Van Boxtel, M. P. J., van der Elst, W., Jolles, J., Verhey, F. R. J., McGlinchey, R. E., Milberg, W. P., & Salat, D. H. (2013). Association between white matter microstructure, executive functions, and processing speed in older adults: The impact of vascular health. Human Brain Mapping, 34(1), 77–95. 10.1002/hbm.21412, 21954054 PMC3830829

[bib26] Kupis, L., Goodman, Z. T., Kornfeld, S., Hoang, S., Romero, C., Dirks, B., Dehoney, J., Chang, C., Spreng, R. N., Nomi, J. S., & Uddin, L. Q. (2021). Brain dynamics underlying cognitive flexibility across the lifespan. Cerebral Cortex, 31(11), 5263–5274. 10.1093/cercor/bhab156, 34145442 PMC8491685

[bib27] Kurtz, M. M., Ragland, J. D., Bilker, W., Gur, R. C., & Gur, R. E. (2001). Comparison of the continuous performance test with and without working memory demands in healthy controls and patients with schizophrenia. Schizophrenia Research, 48(2–3), 307–316. 10.1016/S0920-9964(00)00060-8, 11295383

[bib28] Kurtz, M. M., Ragland, J. D., Moberg, P. J., & Gur, R. C. (2004). The Penn Conditional Exclusion Test: A new measure of executive-function with alternate forms of repeat administration. Archives of Clinical Neuropsychology, 19(2), 191–201. 10.1016/S0887-6177(03)00003-9, 15010085

[bib29] Margulies, D. S., Ghosh, S. S., Goulas, A., Falkiewicz, M., Huntenburg, J. M., Langs, G., Bezgin, G., Eickhoff, S. B., Castellanos, F. X., Petrides, M., Jefferies, E., & Smallwood, J. (2016). Situating the default-mode network along a principal gradient of macroscale cortical organization. Proceedings of the National Academy of Sciences, 113(44), 12574–12579. 10.1073/pnas.1608282113, 27791099 PMC5098630

[bib30] McArdle, J. J., Ferrer-Caja, E., Hamagami, F., & Woodcock, R. W. (2002). Comparative longitudinal structural analyses of the growth and decline of multiple intellectual abilities over the life span. Developmental Psychology, 38(1), 115–142. 10.1037/0012-1649.38.1.115, 11806695

[bib31] McClelland, G. H., Irwin, J. R., Disatnik, D., & Sivan, L. (2017). Multicollinearity is a red herring in the search for moderator variables: A guide to interpreting moderated multiple regression models and a critique of Iacobucci, Schneider, Popovich, and Bakamitsos (2016). Behavior Research Methods, 49(1), 394–402. 10.3758/s13428-016-0785-2, 27531361

[bib32] Misić, B., Mills, T., Taylor, M. J., & McIntosh, A. R. (2010). Brain noise is task dependent and region specific. Journal of Neurophysiology, 104(5), 2667–2676. 10.1152/jn.00648.2010, 20844116

[bib33] Nashiro, K., Sakaki, M., Braskie, M. N., & Mather, M. (2017). Resting-state networks associated with cognitive processing show more age-related decline than those associated with emotional processing. Neurobiology of Aging, 54, 152–162. 10.1016/j.neurobiolaging.2017.03.003, 28390824 PMC5799084

[bib34] Nomi, J. S., Bolt, T. S., Ezie, C. E. C., Uddin, L. Q., & Heller, A. S. (2017). Moment-to-moment BOLD signal variability reflects regional changes in neural flexibility across the lifespan. Journal of Neuroscience, 37(22), 5539–5548. 10.1523/JNEUROSCI.3408-16.2017, 28473644 PMC5452342

[bib35] Nomi, J. S., Spreng, R. N., Bolt, T., Heller, A. S., & Uddin, L. Q. (2019). Test-retest reliability and cognitive relevance of resting-state BOLD signal variability measures. Presented at the Organization for Human Brain Mapping.

[bib36] Nooner, K. B., Colcombe, S., Tobe, R., Mennes, M., Benedict, M. M., Moreno, A. L., Panek, L. J., Brown, S., Zavitz, S. T., Li, Q., Sikka, S., Gutman, D., Bangaru, S., Schlachter, R. T., Kamiel, S. M., Anwar, A. R., Hinz, C. M., Kaplan, M. S., Rachlin, A. B., … Milham, M. P. (2012). The NKI-Rockland Sample: A model for accelerating the pace of discovery science in psychiatry. Frontiers in Neuroscience, 6, 152. 10.3389/fnins.2012.00152, 23087608 PMC3472598

[bib37] Power, J. D., Barnes, K. A., Snyder, A. Z., Schlaggar, B. L., & Petersen, S. E. (2012). Spurious but systematic correlations in functional connectivity MRI networks arise from subject motion. NeuroImage, 59(3), 2142–2154. 10.1016/j.neuroimage.2011.10.018, 22019881 PMC3254728

[bib38] Ragland, J. D., Turetsky, B. I., Gur, R. C., Gunning-Dixon, F., Turner, T., Schroeder, L., Chan, R., & Gur, R. E. (2002). Working memory for complex figures: An fMRI comparison of letter and fractal n-back tasks. Neuropsychology, 16(3), 370–379. 10.1037/0894-4105.16.3.370, 12146684 PMC4332798

[bib39] Rieck, J. R., Rodrigue, K. M., Boylan, M. A., & Kennedy, K. M. (2017). Age-related reduction of BOLD modulation to cognitive difficulty predicts poorer task accuracy and poorer fluid reasoning ability. NeuroImage, 147, 262–271. 10.1016/j.neuroimage.2016.12.022, 27979789 PMC5303662

[bib40] Salthouse, T. A. (2009). When does age-related cognitive decline begin? Neurobiology of Aging, 30(4), 507–514. 10.1016/j.neurobiolaging.2008.09.023, 19231028 PMC2683339

[bib41] Samanez-Larkin, G. R., Kuhnen, C. M., Yoo, D. J., & Knutson, B. (2010). Variability in nucleus accumbens activity mediates age-related suboptimal financial risk taking. Journal of Neuroscience, 30(4), 1426–1434. 10.1523/JNEUROSCI.4902-09.2010, 20107069 PMC2821055

[bib42] Smith, S. M., Beckmann, C. F., Andersson, J., Auerbach, E. J., Bijsterbosch, J., Douaud, G., Duff, E., Feinberg, D. A., Griffanti, L., Harms, M. P., Kelly, M., Laumann, T., Miller, K. L., Moeller, S., Petersen, S., Power, J., Salimi-Khorshidi, G., Snyder, A. Z., Vu, A. T., … Glasser, M. F. (2013). Resting-state fMRI in the Human Connectome Project. NeuroImage, 80, 144–168. 10.1016/j.neuroimage.2013.05.039, 23702415 PMC3720828

[bib43] Son, S. J., Kim, J., Lee, E., Park, J. Y., Namkoong, K., Hong, C. H., Ku, J., Kim, E., & Oh, B. H. (2015). Effect of hypertension on the resting-state functional connectivity in patients with Alzheimer’s disease (AD). Archives of Gerontology and Geriatrics, 60(1), 210–216. 10.1016/j.archger.2014.09.012, 25307953

[bib44] Spear, L. P. (2013). Adolescent neurodevelopment. Journal of Adolescent Health, 52(2 Suppl. 2), S7–S13. 10.1016/j.jadohealth.2012.05.006, 23332574 PMC3982854

[bib45] Spreng, R. N., & Turner, G. R. (2019). The shifting architecture of cognition and brain function in older adulthood. Perspectives on Psychological Science, 14(4), 523–542. 10.1177/1745691619827511, 31013206

[bib46] Uddin, L. Q. (2015). Salience processing and insular cortical function and dysfunction. Nature Reviews Neuroscience, 16(1), 55–61. 10.1038/nrn3857, 25406711

[bib47] Uddin, L. Q. (2020). Bring the noise: Reconceptualizing spontaneous neural activity. Trends in Cognitive Sciences, 24(9), 734–746. 10.1016/j.tics.2020.06.003, 32600967 PMC7429348

[bib48] Vij, S. G., Nomi, J. S., Dajani, D. R., & Uddin, L. Q. (2018). Evolution of spatial and temporal features of functional brain networks across the lifespan. NeuroImage, 173, 498–508. 10.1016/j.neuroimage.2018.02.066, 29518568 PMC6613816

[bib49] von Neumann, J., Kent, R. H., Bellinson, H. R., & Hart, B. I. (1941). The mean square successive difference. Annals of Mathematical Statistics, 12(2), 153–162. 10.1214/aoms/1177731746

[bib50] Waldstein, S. R., Giggey, P. P., Thayer, J. F., & Zonderman, A. B. (2005). Nonlinear relations of blood pressure to cognitive function: The Baltimore Longitudinal Study of Aging. Hypertension, 45(3), 374–379. 10.1161/01.HYP.0000156744.44218.74, 15699446

[bib51] Yan, C.-G., Wang, X.-D., Zuo, X.-N., & Zang, Y.-F. (2016). DPABI: Data processing & analysis for (resting-state) brain imaging. Neuroinformatics, 14(3), 339–351. 10.1007/s12021-016-9299-4, 27075850

