# Supplementary material for: Brain signal variability and executive functions across the life span
Source: Netw Neurosci. 2024 Apr 1;8(1):226–40. doi: 10.1162/netn_a_00347 (PMC10918754; doi:10.1162/netn_a_00347)
Supplement: Supplementary file 1 [file netn-8-1-226-s001.pdf]

Supplemental Table 1

Standardized factor loadings of the modified four-factor model of executive functions.

	Cognitive Flexibility	Inhibitory Control	Working Memory	Processing Speed
TMT B	.79			
CST	.43			
LF	.52			
CF	.58			
CS	.53			
CWI – I		.87		
CWI – S		.87		
N-Back 0			.75	
N-Back 1			.77	
N-Back 2			.68	
CPT RT			.66	
TMT A				.89
TMT MS				.65
PCET RT				.39

Covariances between factors.

	Cognitive Flexibility	Inhibitory Control	Working Memory	Processing Speed
Cognitive Flexibility	-			
Inhibitory Control	.85	-		
Working Memory	.57	.52	-	
Processing Speed	.92	.74	.52	-

Supplemental Table 2

Linear regression coefficients of linear, quadratic, and cubic age terms with each executive function factor.

	Age – Linear			Age – Quadratic			Age – Cubic		
	β	*SE*	*p*	β	*SE*	*p*			
Cognitive Flexibility	-.40	.07	< .001	-.40	.003	< .001	.38	< .001	< .001
Inhibitory Control	-.30	.04	< .001	-.37	.002	< .001	.43	< .001	< .001
Working Memory	-.25	.13	< .001	-.25	.006	< .001	.57	< .001	< .001
Processing Speed	-.43	.03	< .001	-.43	.001	< .001	.35	< .001	< .001

Supplemental Table 3

Simple slopes of significant interactions between network signal variability and age. Age ranges were selected to represent major sections of the lifespan.

	**Adolescence (< 20 years)**			**Early Adulthood (20 – 39 years)**			**Middle Adulthood (40 – 59 years)**			**Late Adulthood (> 60 years)**		
	**β**	***SE***	***p***	**β**	***SE***	***p***	**β**	***SE***	***p***	**β**	***SE***	***p***
Default Mode Network Variability												
Cognitive Flexibility	-.06	.07	.381	.06	.05	.251	**.26**	**.07**	**< .001**	**.43**	**.11**	**< .001**
Inhibitory Control	-.04	.07	.566	.07	.05	.168	**.26**	**.07**	**< .001**	**.42**	**.11**	**< .001**
Working Memory	-.05	.07	.493	.07	.05	.182	**.27**	**.07**	**< .001**	**.43**	**.11**	**< .001**
Processing Speed	-.11	.07	.137	< .01	.06	.983	**.19**	**.08**	**.013**	**.35**	**.12**	**.004**
Central Executive Network Variability												
Cognitive Flexibility	**.16**	**.08**	**.035**	.06	.06	.276	-.10	.08	.201	-.24	.12	.058
Inhibitory Control	.14	.08	.079	.04	.06	.507	-.12	.08	.123	**-.26**	**.13**	**.042**
Working Memory	.14	.08	.059	.05	.06	.392	-.11	.08	.167	-.24	.13	.054
Processing Speed	**.21**	**.08**	**.011**	.08	.06	.211	-.14	.09	.096	**-.33**	**.14**	**.016**
Whole Brain Variability												
Cognitive Flexibility	-.10	.06	.068	< .01	.04	.981	**.17**	**.06**	**.004**	**.31**	**.09**	**< .001**
Inhibitory Control	**-.13**	**.06**	**.020**	-.03	.05	.519	**.15**	**.06**	**.014**	**.29**	**.09**	**.001**
Working Memory	-.08	.06	.185	.02	.04	.643	**.18**	**.06**	**.002**	**.31**	**.09**	**< .001**
Processing Speed	-.02	.06	.734	.02	.05	.722	.08	.06	.201	.13	.10	.164

Note. Bolded values are significant ($p < .050$).

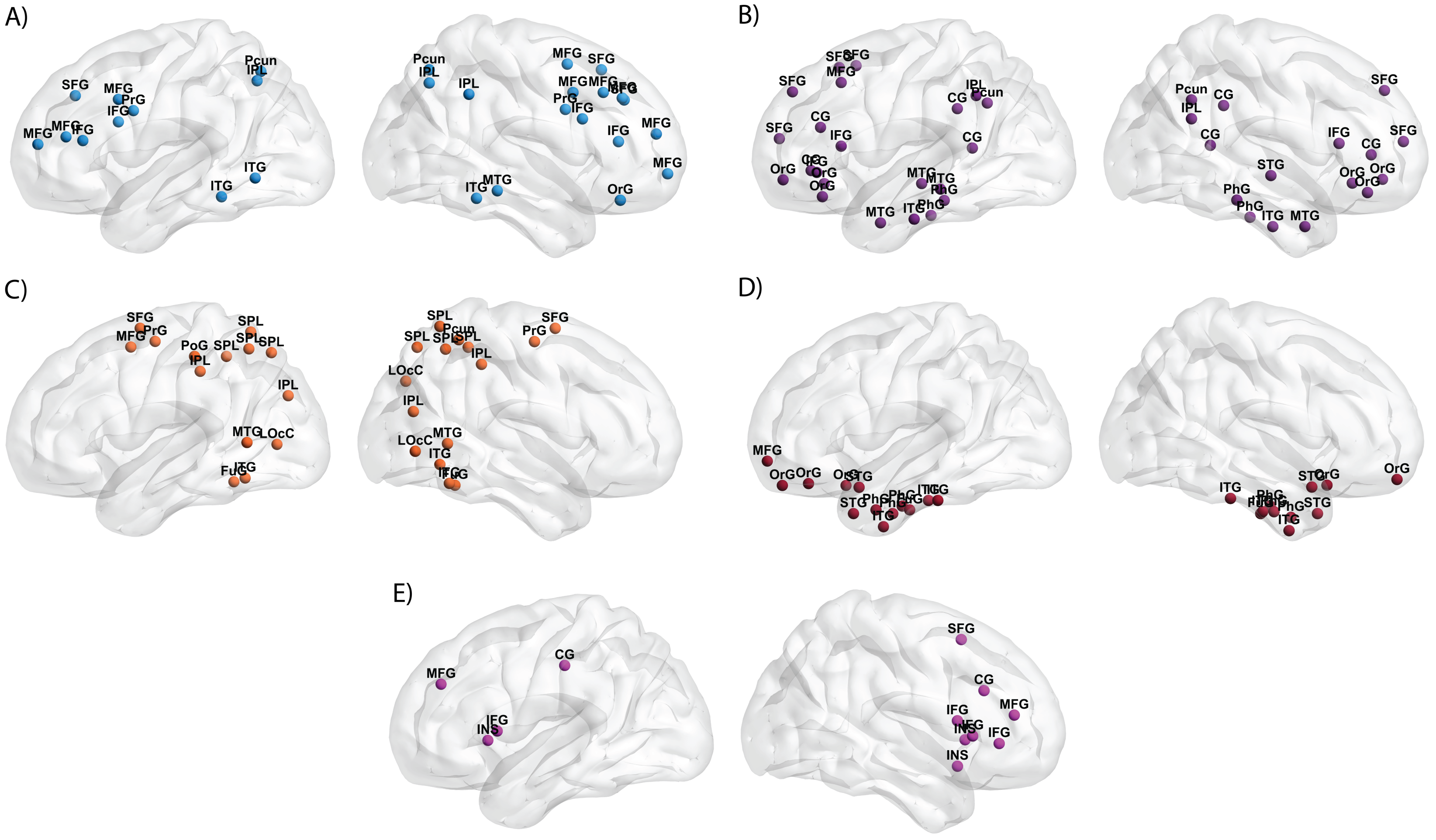

Supplemental Figure 1. ROIs representing each brain network in the Human Brainnetome Atlas (Fan et al., 2016): (A) the Central Executive Network, (B) the Default Mode Network, (C) the Dorsal Attention Network, (D) the Limbic Network, and (E) the Salience Network. SFG = Superior Frontal Gyrus; MFG = Medial Frontal Gyrus; IFG = Inferior Frontal Gyrus; OrG = Orbitofrontal Gyrus; CG = Cingulate Gyrus; PrG = Precentral Gyrus; PoG = Postcentral Gyrus; SPL = Superior Parietal Lobule; IPL = Inferior Parietal Lobule; Pcun = Precuneus; LOcC = Lateral Occipital Cortex; STG = Superior Temporal Gyrus; MTG = Middle Temporal Gyrus; ITG = Inferior Temporal Gyrus; INS = Insular Gyrus. ROIs are visualized with BrainNet Viewer (Xia et al., 2013).

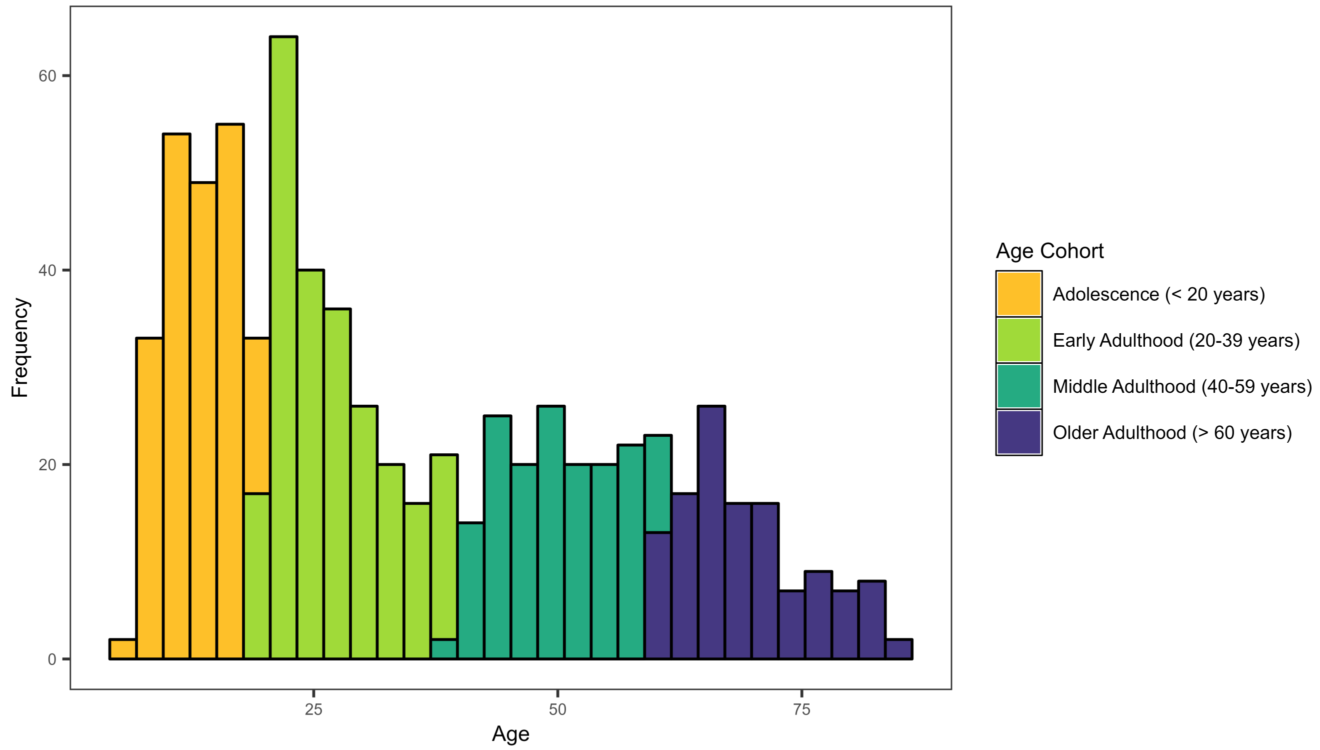

Supplemental Figure 2. Distribution of age across the full sample and by age cohort.
